# Suppressive effects of liquid crystal compounds on the growth of U937 human leukemic monocyte lymphoma cells

**DOI:** 10.1186/1475-2867-12-3

**Published:** 2012-02-03

**Authors:** Junya Ishikawa, Yuuka Takahashi, Masaharu Hazawa, Yukako Fukushi, Atsushi Yoshizawa, Ikuo Kashiwakura

**Affiliations:** 1 Department of Radiological Life Sciences, Hirosaki University Graduate School of Health Sciences, 66-1 Hon-cho, 036-8203, Aomori Hirosaki, Japan; 2 Department of Frontier Materials Chemistry, Graduate School of Science and Technology, Hirosaki University, 3 Bunkyo-cho, Hirosaki 036-8561, Japan

**Keywords:** Liquid-crystalline compound, U937 human leukemic monocyte lymphoma cells, S-phase arrest

## Abstract

**Background:**

The aim of this study was to evaluate the biological and pharmaceutical activities of 14 amphiphilic liquid-crystalline compounds (LCs), i.e, phenylpyrimidine derivatives possessing D-glucamine and cyanobiphenyl derivatives with a terminal hydroxyl unit.

**Results:**

The cytotoxic properties of the LCs on the cell growth, cell cycle distribution, and cell signaling pathway of U937 human leukemic monocyte lymphoma cells were assessed by flow cytometry and western blot analysis. Some LCs showed cytostatic effects, suppressing cell growth via S-phase arrest and without apoptosis in U937 cells. To investigate the mechanisms of the LC-induced S-phase arrest, proteins relevant to cell cycle regulation were investigated by western blot analysis. The rate of LC-induced S-phase arrest was congruent with the decreased expression of MCM2, cyclin A, cyclin B, CDK2, phospho-CDK1 and Cdc25C. Observed changes in cell cycle distribution by LC treated might be caused by insufficient preparation for G2/M transition. Considering the structure of the LCs, the rod-like molecules displaying cytotoxicity against U937 cells possessed flexible spacers with no bulky polar group attached via the flexible spacer.

**Conclusions:**

Our results revealed that some LCs showed cytotoxic properties against non-solid type tumor human leukemic cells via LC-induced S-phase arrest and decreasing expression of several cell cycle related proteins.

## Background

Chemotherapy can regulate the uncontrolled proliferation of abnormal cancer cells by using various types of drugs. The majority of chemotherapeutic drugs can be divided into categories, including the alkylating agents, antimetabolites, anthracyclines, plant alkaloids, topoisomerase inhibitors, monoclonal antibodies, and other antitumor agents [1-7]. Although several types of chemotherapeutic agents have been developed recently, such as molecular targetting drugs, the tyrosine kinase inhibitor Imatinib, only few drugs may result in complete recovery of cancer patients. Therefore, it is essential to develop novel drugs for cancer treatment.

Liquid-crystalline compounds (LCs) are widely used in display media in televisions and personal computers. LCs are classified into various categories on the basis of their structural characteristics. One of the principal compounds is an amphiphilic compound, consisting of hydrophobic and hydrophilic components. Amphiphilic liquid crystals are thought to have structural affinity to the cell membranes, which are lamellar bilayer mesophases of phospholipids, glycolipids, and cholesterol. Therefore, some lyotropic LCs displaying a structural affinity to the cell membranes have been applied for the development of novel drug delivery systems [3]. Although these amphiphilic LCs seem promising for biological applications, the pharmacological properties of LCs are not well understood, and therefore, must be elucidated.

Our recent reports demonstrated that some lyotropic LC materials, namely, the phenylpyrimidine and cyanobiphenyl derivatives, showed cytostatic effects on the growth of solid tumor A549 human lung cancer cells, causing G1-phase arrest in cells. One of the phenylpyrimidine derivatives inhibited A549 growth without any toxicity to normal fibroblasts [8,9]. However, it is not yet known whether these LCs have cytotoxic properties against non-solid type tumor leukemic cells that are commonly treated by chemotherapy. To clarify this issue, we investigated the cytotoxic properties of 14 amphiphilic LCs against the human leukemic monocyte lymphoma cell line U937.

## Results

### Screening of LCs with respect to the cytotoxicity against U937 cells

**Figure 1:**
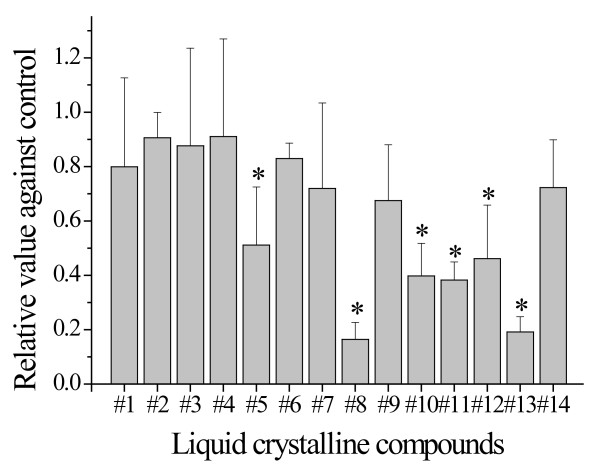
**Screening of LCs with respect to the cytotoxicity against U937 cells**. Effects of liquid crystalline compounds on the growth of theU937 human leukemic monocyte lymphoma cell line. The cells were cultured in liquid medium containing 10 μM of each compound for 48 h. *p < 0.05 using Student's *t*-test.

The effect of each compound (10 μM) on the growth of the U937 cells was tested to investigate the cytotoxic properties of the LCs shown in Table 1. Compound #8, which contains a cyanobiphenyl in its central position, and compound #13, which contains a phenylpyrimidine, showed the maximum suppressive effect among all the compounds tested herein (Figure 1). Therefore, subsequent studies were performed using compounds #8 and #13 to characterize their suppressive mechanisms on the growth of U937 cells.

**Table 1:** Structure of liquid crystalline compounds

Sample Number	Structural formula
#1	
#2	
#3	
#4	
#5	
#6	
#7	
#8	
#9	
#10	
#11	
#12	
#13	
#14	

**Figure 2:**
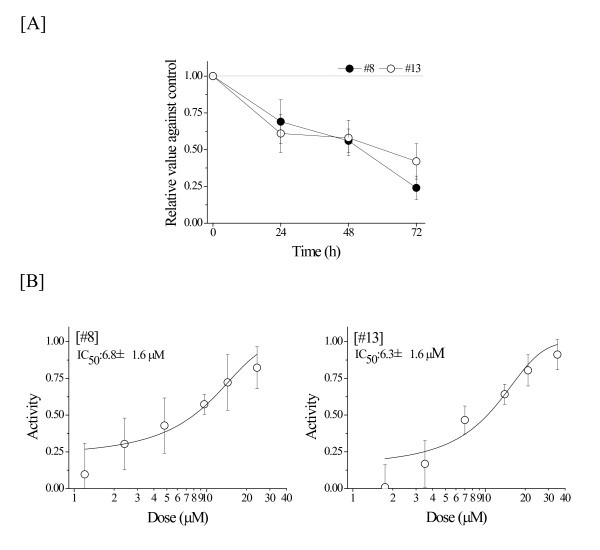
**Suppressive effects of the liquid crystalline compounds #8 and #13 on the growth of U937 cells**. [A] Cells were treated with 6-7 μM liquid crystalline compounds or DMSO for the indicated periods. [B] The dose response curves of U937 cells to liquid crystalline compounds. Activity shows growth inhibition rate. The IC_50 _values were determined using the Boltzmann functions. Values are the mean ± standard deviation (S.D.) value of more than 3 separate experiments in duplicate wells.

To determine the growth inhibitory profiles for #8 and #13 on U937 cells, proliferation assays were performed at various time points and concentrations. Both compounds #8 and #13 exhibited cytotoxic properties in a time-dependent manner (Figure 2A). The IC_50 _values of compounds #8 and #13 were 6.8 and 6.3 μM, respectively (Figures 2B, C). Therefore, subsequent experiments were mainly performed using the determined IC_50 _values.

### Effects of LCs on apoptosis and cell cycle distribution

**Figure 3:**
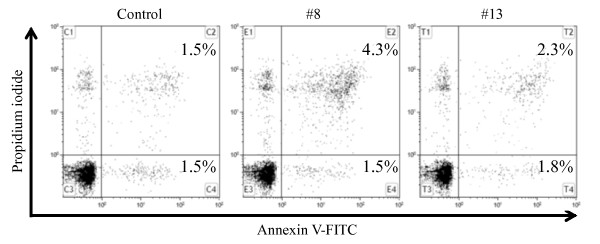
**Analysis of apoptosis in U937 cells**. The cells were treated with IC_50 _value of the compounds #8 and #13 or DMSO for 48 h. The x-axis indicates the Annexin V-positive populations, and the y-axis indicates the propidium iodide-positive populations.

During the early stages of apoptosis, translocation of phosphatidylserine (PS) to the cell surface is a key signal in the apoptotic pathway. Annexin V is a Ca^2+^-dependent phospholipid-binding protein with high affinity for PS [10]. Therefore, to elucidate whether apoptotic induction is involved in the cytotoxic property of LCs, cells were analyzed by flow cytometry using Annexin V-FITC and PI staining (Figure 3). Although each of the LCs induced 50% growth suppression, indiscernible changes were observed between the control and LC-treated groups in each fraction (each Annexin V-positive/PI-negative fraction of control, #8 and #13 was 1.5% and 1.8%, respectively). These data indicate that the cytotoxic properties of LCs are not related to apoptotic and necrotic induction of cell death.

### S-phase arrest by LCs in the cell cycle of U937 cells

**Figure 4:**
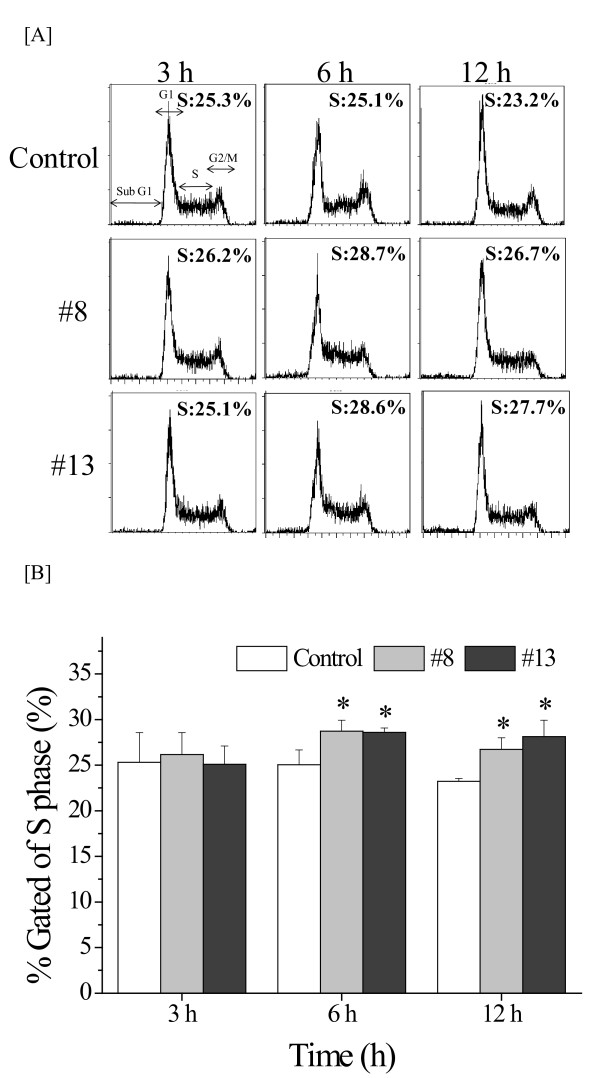
**Effect of liquid crystalline compounds on the cell cycle distribution in U937 human leukemic monocyte lymphoma cells**. [A] Flow cytometry analysis of U937 cells treated with each compounds for indicated times. Representative cytograms are shown. [B] The rate of S-phase in U937 cells was shown. Values are the mean ± standard deviation (S.D.) value of at least 3 separate experiments. *p < 0.05 by Student's *t*-test.

Since anti-tumor drugs usually affect the cell cycle distribution [11], the cell cycle distribution in U937 cells treated with LCs was analyzed by flow cytometry. As shown in Figure 4A, no modification of the cell cycle distribution was observed in LCs-treated cells compared to the controls at 3 h. However, LCs significantly induced the S-phase population at 6 and 12 h (Figure 4B). These data indicated that LCs induced S-phase arrest in U937 cells.

### Inhibitory regulation of several proteins expression related to cell cycle progression by LCs in U937 cells

**Figure 5:**
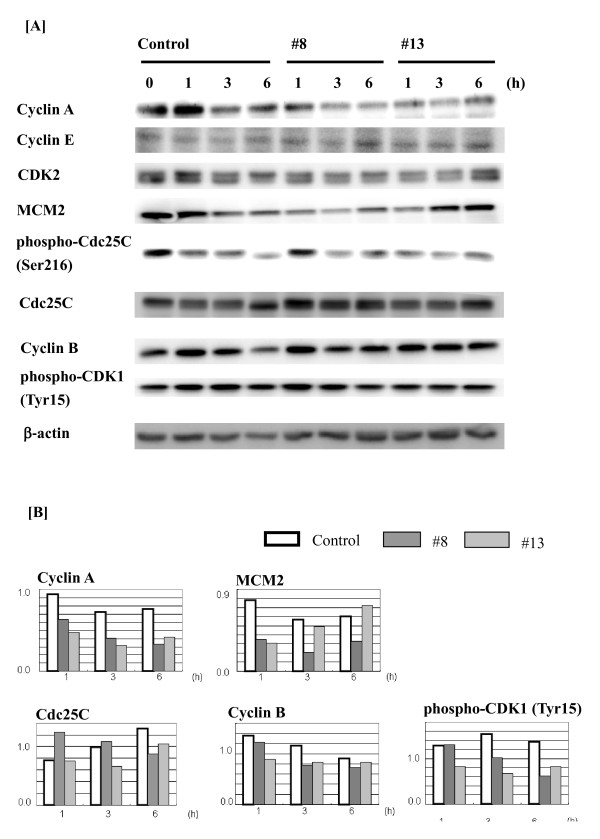
**Western blot profiles of cell cycle-related molecules in the U937 cells**. [A and B] Western blot profiles of cell cycle-related molecules in the U937 cells treated with IC_50 _value of the compounds #8 and #13 for 0-6 h. β-actin is the loading control. Immunoblotting for each protein was performed at least twice, with comparable results.

To investigate the mechanism of S-phase arrest induction by LCs, proteins relevant to cell cycle regulation were evaluated by western blot analysis of U937 cells treated with compounds #8 and #13 (Figure 5). CDK2 and cyclin E are known to regulate progression through G1/S transition [12,13], and MCM2 is required in the acquisition of DNA replication fork [14,15]. Cyclin A is required for both progression S phase and M phase [16]. Activation of CDK1 regulate entry into mitosis, and is controlled at several steps including cyclin B binding and dephosphorylation of CDK1 at Thr 14/Tyr15 by Cdc25C phosphatase [17-19]. As shown in Figure 5, the expression of MCM2 was decreased by LC treatment at 1 h, and the expression of cyclin A, cyclin E, CDK2 were decreased by LC treatment at 1-6 h. In addition, the expression of Cdc25C was decreased by LC treatment at 6 h, and the expression of phospho-CDK1 (Tyr 15) was decreased by LC treatment at 1-6 h.

## Discussion

The pharmaceutical activities of 14 amphiphilic LCs were examined in human leukemic monocyte lymphoma U937 cells (Table 1). Some LCs (#5, #8, #10-13) showed cytotoxic properties (Figure 1). Among the LCs tested, compounds #8 and #13 showed marked cytotoxicity, and the IC_50 _values of these compounds were similar (6.8 and 6.3 μM, respectively) (Figure 2). However, cytotoxic effects by these compounds had no relation to apoptosis induction supported by Annexin V/PI staining analysis (Figure 3). Both compounds were considered to delay S-phase progression in cell cycle, through inhibition of MCM2, cyclin A, cyclin B, CDK2, phospho-CDK1 (Tyr15) and Cdc25C expression (Figures 4, 5).

The cell cycle is a fundamental and ordered event in which DNA replicates, and homologous chromosomes segregate and equally distributed among the daughter cells. Therefore, the oscillation of specific protein expressions is important in cell cycle progression. The tyrosine phosphatase Cdc25C serve as mitotic activator through dephosphorylation of CDK1 at Tyr14/15 and is negatively regulated by phosphorylation at Ser216. Because LCs is considered to prevent S-phase progression through MCM2 inhibition and insufficient cyclin proteins, decreased phospho-CDK1 (Tyr15) might reflect the failure of Tyr15 phosphorylation by Wee1 in G2 phase. Therefore, majority of CDK1 exist as inactivated form comparing to control and both Cdc25C and phos-Cdc25C (Ser216) decrease imply mere lack of preparation for cell cycle progression. Similar phenomenon were reported that reducing cyclin A/CDK2 and Cdc25C expressions lead to S phase arrest in human Lovo colon cancer cells [19].

The cytotoxic property and classes of active compound depend on cell types because compounds cytotoxic to U937 cells (#5, #8, #10-13) are not necessarily cytotoxic to other cell types, lung cancer A549 cells [8]. Compounds #5, #10, #12, and #13 had little effects against A549 cells and compound #8 induced not S-phase arrest observed present study but G1-phase arrest in A549 cells without apoptosis [8]. Nevertheless, apoptosis unrelated toxicity is common feature of compound #8, which is supporsed torelate with p53 independent mechanism because A549 and U937 cells have valid p53 genomic contexts [20,21]. The loss of p53 function generally leads to resistance to chemotherapeutic drugs, tumor malignancy and metastasis [22], therefore, compound #8 could be an effective drug against a variety of tumors regardless of p53 status.

The structure of LCs may be responsible for the cell-specific cytotoxic effects exerted by the different LCs. For example, #11, in which D-glucamine and phenylpyrimidine are connected via a flexible spacer, displayed a strong cytotoxic effect against A549 cells, but showed only a moderate cytotoxic effect against U937 cells. Compound #13, in which D-glucamine was removed from #11, did not exert cytotoxic effects against A549 cells; however, it showed a strong cytotoxic effect against U937 cells. These results suggest that differences in the molecular structures of the LCs were responsible for the differences in their cell type-specific inhibitory effect. Previously, Takahashi et al. demonstrated that the suppressive effects of cyanobiphenyl derivatives on A549 cells depended on alkyl chain length rather than on the terminal substrate or the position of hydroxyl moieties [8]. Interestingly, both compounds #8 and #13, which showed strong cytotoxic effects against U937 cells, possess -O(CH_2_)_6_OH in their structure. Furthermore, the compounds that had cytotoxic activity against U937 cells (#5, #8, #10, #11, and #13) were structured as rod-like molecules. These rod-like molecules possess a flexible spacer consisting of an alkyl chain such as (CH_2_)_6 _and are not attached to a bulky polar group such as -OH, -CN, and -COOH via this flexible spacer. These structures might play an important role in the cytotoxic effects against U937 by LCs. Further, studies are necessary to elucidate the structure-activity relationships.

It remains unclear how active LC compounds inhibited expression of several cell cycle related proteins. However, LCs clearly suppressed the growth of tumor cells independent of p53. In addition, the cytotoxicity of compound #8 is enhanced by mixing another LC at an equimolar concentration, which is suspected to result in an antiparallel molecular alignment via a dipole-dipole interaction [23]. Because anti-cancer drugs used at clinical situation remains several problems including pharmacological tolerance and secondary effect, further studies are clearly required to elucidate LC's structural properties and its pharmacological activity.

## Conclusion

In this study, some LCs showed cytostatic effects, suppressing cell growth via S-phase arrest and without apoptosis in U937 cells. Furthermore, LCs clearly suppressed the growth of tumor cells independent of p53. Our results suggest that some LCs possessing cytotoxic properties may be useful for cancer treatments irrespective of their p53 status.

## Methods

### Liquid crystal compounds

Compound #1 was provided by Midori Kagaku Co., Ltd. (Tokyo, Japan). Compound #2 was provided by Japan Energy Corporation (Tokyo, Japan) and compound #3 was purchased from the Kanto Chemical Co., Ltd. (Tokyo, Japan). The other compounds in Table 1 were synthesized in our laboratory.

Each compound was dissolved in dimethyl sulfoxide (DMSO; Wako, Osaka, Japan) at 0.1 mM, 1 mM, and 10 mM concentrations, respectively. Each dissolved compound was stored at 4°C in the dark.

### Antibodies

Anti-minichromosome maintenance (MCM2) antibody (Rabbit, #2901-1), anti-cell division cycle 25 C (Cdc25C) antibody (Rabbit, #1302-1), and anti-phospho Cdc25C (Ser216) antibody (Rabbit, #1190-1) were purchased from Epitomics Inc. (California, USA). Anti-actin antibody (Goat, C-11, sc-1615) and anti-cyclin dependent kinase 2 (CDK2) antibody (Rabbit, sc-163) were purchased from Santa Cruz Biotechnology (Santa Cruz, California, USA). Anti-cyclin E antibody (Mouse, #4129), anti-cyclin A (BF683) antibody (Mouse, #4656), anti-cyclin B1 (V152) antibody (Mouse, #4135), and anti-phospho cdc2 (Tyr15) antibody (Rabbit, #9111S) were purchased from Cell Signaling Technology (Tokyo, Japan).

### Cell line

The U937 human leukemic monocyte lymphoma cell line was purchased from the RIKEN Bio-Resource Center (Tsukuba, Japan). This cell was maintained in continuous culture in RPMI1640 medium (Gibco^®^, Invitrogen, California, USA) supplemented with 10% heat-inactivated fetal bovine serum (Bioserum, UBC, Japan) in a humidified atmosphere at 37°C and 5% CO_2_.

### Cell growth inhibition assay

In the first screening, the U937 cells were seeded in a 24-well tissue culture plate (Falcon, Becton Dickinson Biosciences, Franklin Lakes, USA) with 500 μl of culture medium at a concentration of 5 × 10^4 ^cells/well, and each LC was added to the culture at a concentration of 10 μM. After 48 h incubation, cells at each condition were harvested, and diluted in trypan blue (Nacalai Tesque, Kyoto, Japan), which distinguishes viable cells from the damaged or dead ones. Cell growth was investigated by microscopy, and counting of viable cells was conducted using Burker-Turk hemocytometer (SLGC, Saitama, Japan). The relative value normalized to control values was calculated as the ratio of the number of LC-treated cells to the number of control cells. In the determination of 50% inhibitory concentration (IC_50_), the U937 cells were seeded in a 48-well tissue culture plate (Falcon) with 200 μl of culture medium at a concentration of 1 × 10^4 ^cells/well, and each LC was added to the culture at various concentrations ranging from 1.5 to 30 μM. After 48 h incubation, cells at each condition were harvested, and the number of viable cells was counted by the trypan blue exclusion test under a microscope. The relative value normalized to control values was calculated as the ratio of the number of LC-treated cells to the number of control cells.

### Detection of apoptosis by flow cytometry

The extent of apoptosis was determined by Annexin V-FITC and propidium iodide (PI) staining according to the manufacturer's instructions. In brief, the cells were suspended with 100 μl of binding buffer (10 mM HEPES/NaOH, 140 mM NaCl, 2.5 mM CaCl_2_; pH 7.4) and stained with Annexin V-FITC for 10 min at room temperature in the dark. After washing with binding buffer, the cells were re-suspended in binding buffer containing PI (20 μg/ml). Apoptotic cells were determined using the Cell Lab Quanta™ SC MPL apparatus. In the Annexin V/PI quadrant gating, AnnexinV(-)/PI(-), AnnexinV(-)/PI(+), AnnexinV(+)/PI(-), and AnnexinV(+)/PI(+) represented the fraction of viable cells, necrotic cells, early apoptotic cells, and late apoptotic/secondary necrotic cells, respectively.

### Cell cycle analysis by flow cytometry

The U937 cells were treated with each of the LCs in a tissue culture dish (Iwaki, Chiba, Japan), according to the method described above. After 3, 6, and 12 h of treatment, the harvested cells were treated with PBS containing 0.1% Triton X-100 (Wako) and stained with propidium iodide (50 μg/ml; Sigma). An analysis of the cell cycle distribution was performed using a flow cytometer (Quanta SC MPL; Beckman-Coulter, Fullerton, California, USA).

### SDS-PAGE and western blot analysis

SDS-PAGE and western blot analysis were performed as described in previous reports [24]. Briefly, U937 cells were treated with each compound in the medium and incubated for the indicated times. The harvested cells were lysed with 50 mM Hepes-HCl (pH 7.4), 100 mM NaCl, 1% Triton X-100, and 1 mM PMSF (Wako) on ice for 30 min, and sonicated twice for 30 s at ice-cold temperature. After centrifugation at 12,000 rpm for 20 min at 4°C, the protein concentration in the supernatant was determined with a Bio-Rad Protein Assay Kit (Bio-Rad Lab, Hercules, California, USA) using a SmartSpec™ Plus (Bio-Rad). Each protein lysate was mixed with 2 × sample buffer (625 mM Tris-HCl, pH6.8, 20% SDS, 2% 2-mercaptoethanol, 2% glycerol). Each sample (30-50 μg/lane) was separated by SDS-PAGE after boiling for 5 min. Next, the proteins in the gel were transferred to a nitrocellulose membrane (Toyo Roshi, Tokyo, Japan). The membrane was reacted with each primary antibody in Tris-buffered saline (10 mM Tris-HCl, pH 7.4, 100 mM NaCl, 0.1% Tween-20), and supplemented with 5% BSA or milk for 1 h at room temperature. After exposure to the primary antibody, membranes were labeled with donkey anti-rabbit IgG-HRP or anti-goat IgG-HRP antibody (Santa Cruz) or anti-mouse IgG-HRP antibody (R and D Systems, Minneapolis, USA) and the antigens were detected using ECL Plus Western Blotting Detection Reagents (GE Healthcare Japan, Tokyo, Japan).

### Statistical analysis

The significance of the differences between the control and experimental groups were determined by Student's *t-*test or Mann-Whitney's *U*-test depending on the distribution. Statistically significant differences were defined as p-values of less than 0.05.

## Abbreviations

LCs: liquid-crystalline compounds.

## Competing interests

The authors declare that they have no competing interests.

## Authors' contributions

JI and MH were responsible for performing the majority of assays, discussing the study design and manuscript writing. YT and YF were responsible for performing a portion of the studies. AY was responsible for conceptualization of study ideas, study design, synthesization of liquid crystal compounds. IK was responsible for the entire project including conceptualization of study ideas, study design, evaluating results, study discussion, data analysis, manuscript writing and funding the study. All authors read and approved the final manuscript.
